# Scleroderma-Like Phenotype in a Patient With Transthyretin Cardiac Amyloidosis and Acromegaly: A Triple Diagnostic Challenge

**DOI:** 10.7759/cureus.117024

**Published:** 2026-09-27

**Authors:** Ayesha Javed, Paige Debuys, Kim Nguyen

**Affiliations:** 1 Internal Medicine, Mercy Health System, Fairfield, USA; 2 Rheumatology, TriHealth, Cincinnati, USA

**Keywords:** acromegaly, diagnostic dilemma, pituitary macroadenoma, systemic sclerosis, transthyretin amyloidosis

## Abstract

Systemic sclerosis, transthyretin cardiac amyloidosis, and acromegaly are rare disorders that share several clinical manifestations, including skin thickening, heart failure, dysphagia, fatigue, and soft tissue enlargement. Their coexistence is exceptionally rare and poses a significant diagnostic challenge, increasing the risk of premature diagnostic closure.

We report a 78-year-old man who developed progressive diffuse skin thickening following spinal cord stimulator revision. Positive RNA polymerase III antibodies supported a diagnosis of diffuse systemic sclerosis. Evaluation of worsening edema and exertional dyspnea revealed severe concentric left ventricular hypertrophy with relative apical sparing on echocardiography, and technetium-99m pyrophosphate scintigraphy confirmed transthyretin cardiac amyloidosis. Persistent macroglossia, coarse facial features, prior carpal tunnel syndrome, and a remote history of increasing ring and shoe size prompted endocrine evaluation, which demonstrated elevated insulin-like growth factor-1 levels, failure of growth hormone suppression, and a pituitary macroadenoma consistent with acromegaly. The patient underwent successful transsphenoidal resection with biochemical remission.

This case highlights the importance of reconsidering the differential diagnosis when clinical features are not fully explained by an established diagnosis. Recognizing atypical and discordant findings prevented premature diagnostic closure and led to the identification of transthyretin cardiac amyloidosis and acromegaly in a patient with a rapidly progressive scleroderma-like phenotype.

## Introduction

Systemic sclerosis is a chronic autoimmune connective tissue disease characterized by immune dysregulation, widespread vasculopathy, and progressive fibrosis of the skin and internal organs. Diffuse cutaneous systemic sclerosis is the more aggressive subtype, typically presenting with rapidly progressive skin thickening and early involvement of visceral organs, including the lungs, gastrointestinal tract, kidneys, and heart. Disease-specific autoantibodies, particularly RNA polymerase III antibodies, are highly specific for systemic sclerosis and play an important role in supporting the diagnosis and defining the disease phenotype when interpreted within the appropriate clinical context [1]. Transthyretin amyloidosis results from the extracellular deposition of misfolded transthyretin-derived amyloid fibrils. Cardiac involvement typically manifests as restrictive cardiomyopathy with progressive diastolic dysfunction, left ventricular wall thickening, conduction abnormalities, and atrial or ventricular arrhythmias. Common extracardiac manifestations include bilateral carpal tunnel syndrome, lumbar spinal stenosis, and peripheral neuropathy, often preceding cardiac disease by several years [2]. Acromegaly is a rare endocrine disorder caused by a growth hormone-secreting pituitary adenoma. Chronic excess growth hormone leads to elevated insulin-like growth factor-1 (IGF-1) levels, resulting in progressive overgrowth of bone and soft tissues [3]. Because the disease develops insidiously, the diagnosis is often delayed for years after symptom onset. Common clinical manifestations include enlargement of the hands and feet, coarse facial features, macroglossia, carpal tunnel syndrome, arthropathy, hypertension, obstructive sleep apnea, and cardiomyopathy [4].

Although these disorders have distinct pathophysiologic mechanisms, their overlapping clinical manifestations can obscure the diagnosis when they occur concurrently. Distinguishing features may be subtle, making careful reassessment of discordant findings essential. In particular, acromegaly and other scleroderma-like disorders may complicate the interpretation of skin and soft tissue changes in patients being evaluated for systemic sclerosis. The coexistence of these three disease processes presents a particularly challenging diagnostic scenario because of their overlapping clinical manifestations. We describe a patient in whom persistent attention to unexplained clinical findings prevented premature diagnostic closure and led to the recognition of overlapping disease processes requiring distinct diagnostic and therapeutic approaches.

## Case presentation

A 78-year-old man with a history of severe lumbar spinal stenosis requiring laminectomy and spinal cord stimulator placement underwent revision of the stimulator in April 2021. The procedure was complicated by intraoperative hypotension and bradyarrhythmia requiring intubation. In the months following surgery, he developed progressive skin thickening beginning on the dorsum of the right hand and subsequently extending to both upper extremities, lower extremities, abdomen, and back. Although temporally associated with the spinal intervention, no causal relationship was established. Associated symptoms included fatigue, diffuse arthralgias, dysphagia, intermittent bilateral lower extremity edema, and a pruritic rash.

Dermatologic evaluation with punch biopsy was initially interpreted as morphea/scleroderma-like change and demonstrated prominent dermal sclerosis with markedly thickened, sclerotic collagen. Given the atypical presentation, dermatopathologic review considered nephrogenic systemic fibrosis; however, the expected prominent fibroblastic proliferation was absent. Congo red staining and a subsequent skin biopsy showed no evidence of cutaneous amyloid deposition.

On rheumatologic examination, the patient demonstrated marked, symmetric skin thickening and induration of both hands extending proximally beyond the metacarpophalangeal joints to the forearms and elbows, with additional involvement of the feet and lower extremities. The skin was shiny, tight, and markedly indurated without discrete plaques. Bilateral enlargement and swelling of the hands and feet were also noted (Figure 1). There was no obvious sclerodactyly, telangiectasia, or digital ulceration, and the patient denied Raynaud phenomenon.

**Figure 1:**
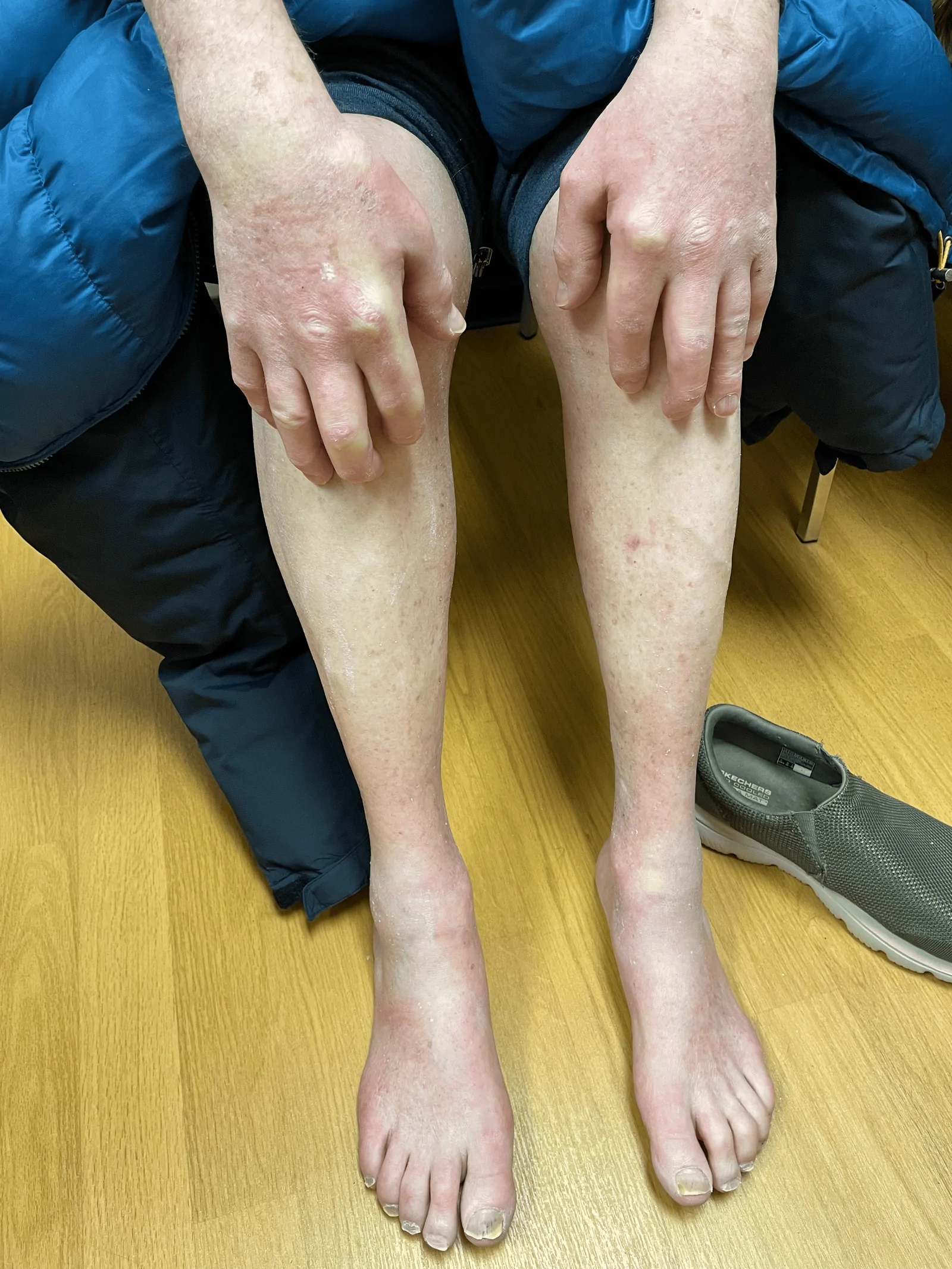
Bilateral enlargement and swelling of the hands and feet

Initial laboratory evaluation revealed negative antinuclear antibody, anti-Scl-70, anti-SSA, anti-SSB, anti-Smith, anti-RNP, anti-double-stranded DNA antibodies, rheumatoid factor, cyclic citrullinated peptide antibodies, and serum and urine monoclonal protein studies. Repeat serologic testing demonstrated RNA polymerase III antibody positivity. Under the 2013 American College of Rheumatology (ACR)/European Alliance of Associations for Rheumatology (EULAR) classification criteria, skin thickening of the fingers extending proximal to the metacarpophalangeal joints fulfills the sufficient criterion for systemic sclerosis classification (9 points); RNA polymerase III antibody positivity contributes an additional 3 points. A modified Rodnan skin score and nailfold capillaroscopy findings were not available. Relevant laboratory findings are summarized in Table 1.

**Table 1:** Laboratory findings during the diagnostic evaluation

Laboratory test	Patient value	Reference value
Antinuclear antibody	Negative	Negative
Anti-Scl-70 antibody	Negative	<1 AI
RNA polymerase III antibody	>80	<20 EZ
Anti-SSA/SSB antibodies	Negative	<1 AI
Anti-Smith antibody	Negative	<1 AI
Anti-RNP antibody	Negative	<1 AI
Anti-dsDNA antibody	<1 IU/ml	<4 IU/ml
Rheumatoid factor	<15 IU/ml	0-14 IU/ml
Anti-CCP antibody	Negative	<20 U/ml
Serum creatinine	0.6 mg/dL	0.8-1.3 mg/dl
Estimated glomerular filtration rate	94 mL/min/1.73 m²	≥60 mL/min/1.73 m²
Serum/urine immunofixation	Negative	No monoclonal protein

Chest computed tomography (CT) demonstrated mild bibasilar interstitial changes. The patient also had recurrent dysphagia requiring esophageal dilation; however, in the absence of esophageal manometry, this was not considered definitive evidence of systemic sclerosis gastrointestinal involvement. Taken together, the rapidly progressive cutaneous sclerosis, supportive histopathology, fulfillment of the 2013 ACR/EULAR classification criteria [5], and RNA polymerase III antibody positivity strongly favored systemic sclerosis. However, the absence of typical clinical features and unavailable nailfold capillaroscopy warranted a cautious interpretation as a scleroderma-like phenotype suggestive of systemic sclerosis.

Prednisone was initiated at 10 mg daily for two weeks, followed by 5 mg daily, without improvement in skin thickening, and was subsequently discontinued. Given RNA polymerase III antibody positivity and corticosteroid exposure, blood pressure and renal function were monitored for possible scleroderma renal crisis. Blood pressure remained well controlled on losartan, with a serum creatinine of 0.6 mg/dL and an estimated glomerular filtration rate of 94 mL/min/1.73 m². Urinalysis was unremarkable except for mild proteinuria that remained stable, and no clinical evidence of scleroderma renal crisis developed during follow-up.

Given the patient's advanced age, rapidly progressive cutaneous sclerosis, and RNA polymerase III antibody positivity, occult malignancy and a possible cancer-associated scleroderma phenotype were also considered [1]. CT imaging of the chest, abdomen, and pelvis identified a renal lesion that showed no concerning activity on subsequent positron emission tomography (PET) imaging. Serum protein electrophoresis and immunofixation were negative, and age-appropriate malignancy screening was unrevealing. No occult malignancy was identified during the available follow-up.

Given progressive exertional dyspnea and worsening lower extremity edema, cardiac evaluation was pursued. Transthoracic echocardiography demonstrated reduced basal and mid-ventricular longitudinal strain with relative preservation of apical strain (Figure 2). The corresponding longitudinal strain bull's-eye map demonstrated the characteristic "cherry-on-top" apical-sparing pattern, raising concern for cardiac amyloidosis (Figure 3). Serum free light chains and serum and urine immunofixation electrophoresis remained negative, making light-chain amyloidosis unlikely. Technetium-99m pyrophosphate scintigraphy demonstrated grade 3 myocardial tracer uptake with a heart-to-contralateral ratio greater than 1.5, supporting the diagnosis of transthyretin cardiac amyloidosis. Echocardiography also demonstrated a speckled appearance of the left ventricular myocardium (Figure 4). Tafamidis 61 mg daily was initiated and continued at follow-up due to good tolerability and favorable clinical response. Low-dose torsemide 10 mg daily was added for lower extremity edema.

**Figure 2:**
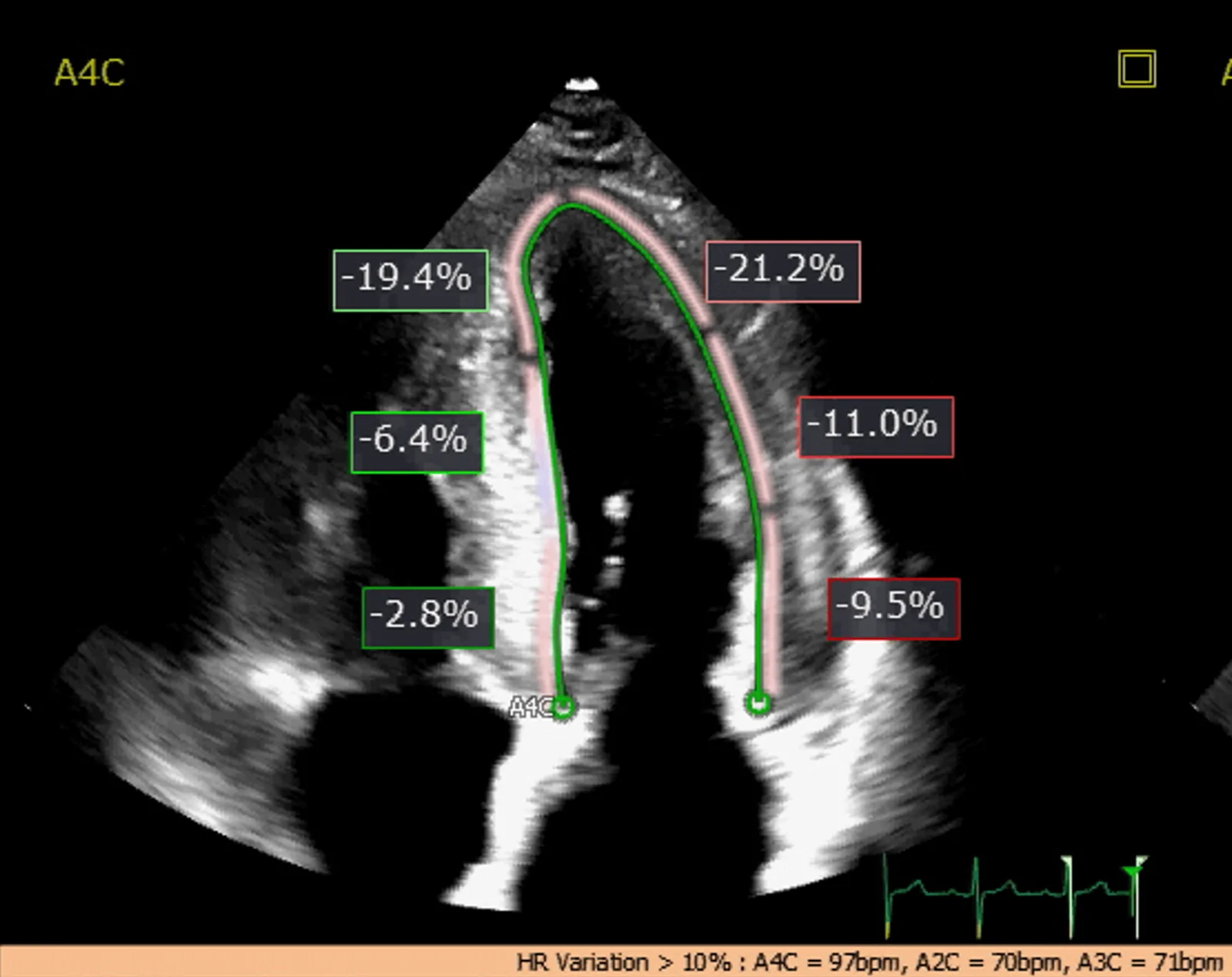
Apical sparing on speckle-tracking echocardiography Apical four-chamber longitudinal strain imaging demonstrates relatively preserved apical strain with reduced mid and basal segment strain, consistent with a relative apical-sparing pattern suggestive of cardiac amyloidosis.

**Figure 3:**
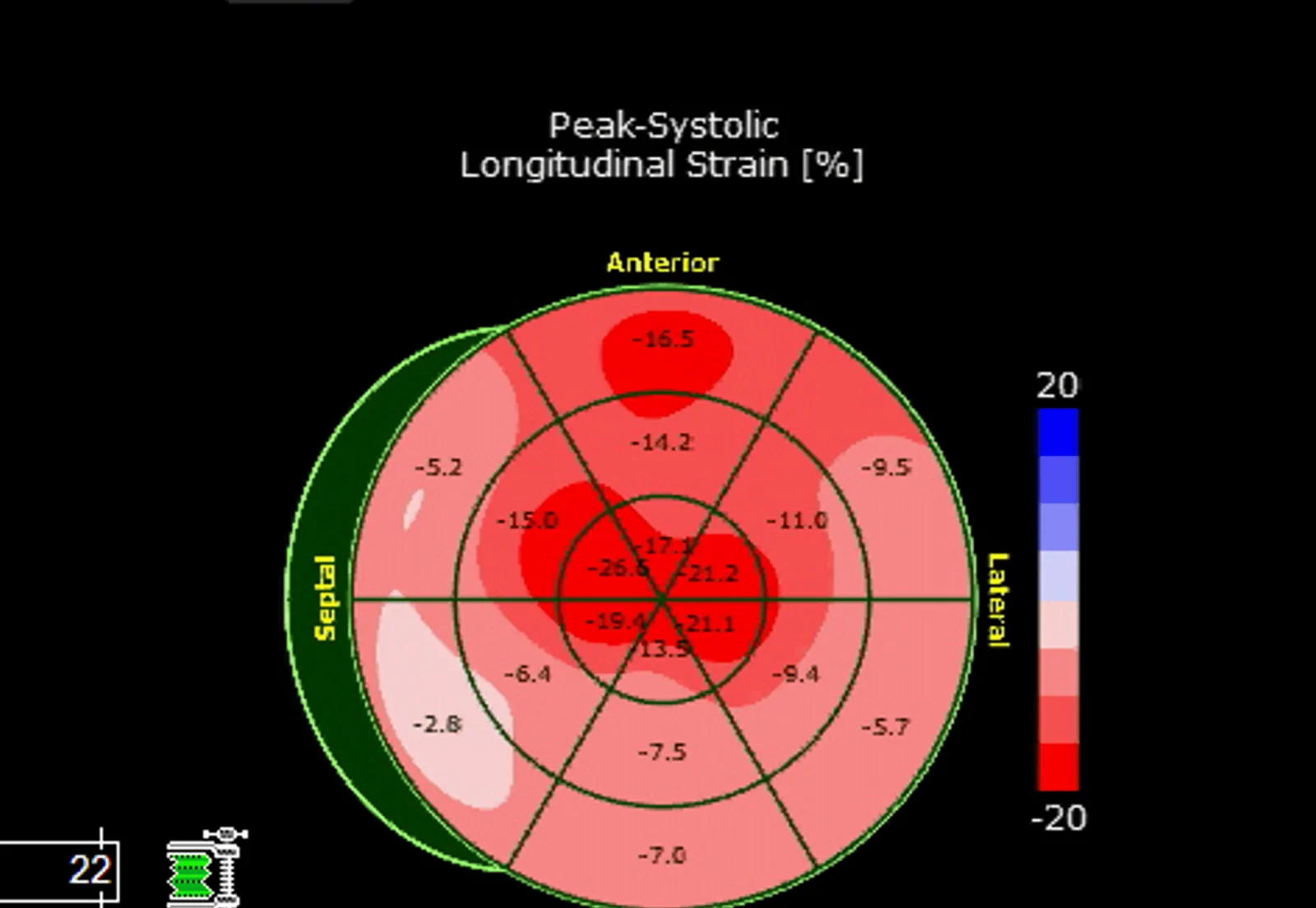
Relative apical-sparing ("cherry-on-top") pattern on longitudinal strain imaging

**Figure 4:**
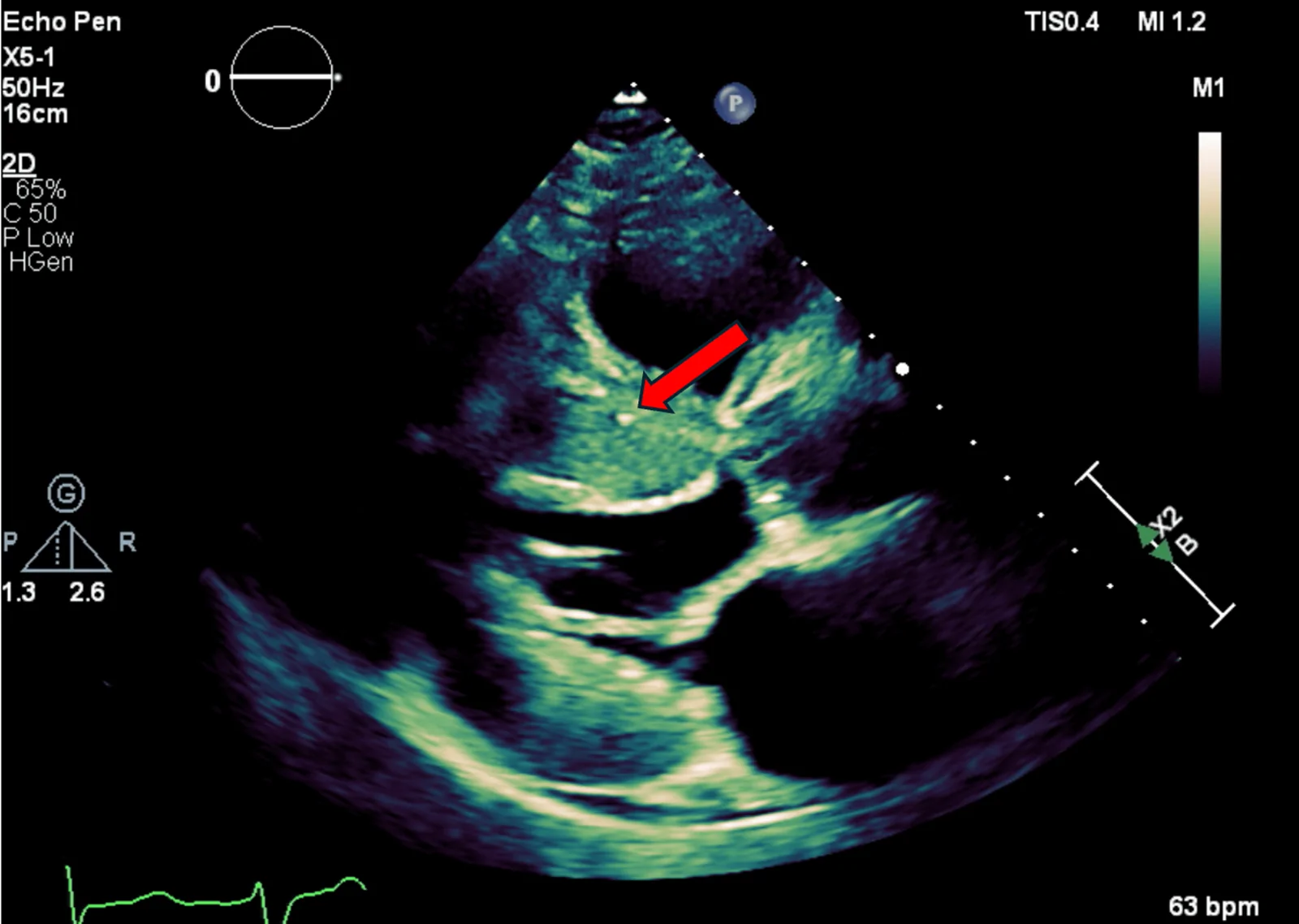
Speckled/ground-glass appearance of left ventricular myocardium (basal anteroseptum)

Although the coexistence of systemic sclerosis and transthyretin cardiac amyloidosis explained many aspects of the patient's clinical presentation, several physical findings remained atypical. Examination demonstrated marked macroglossia and coarse facial features. Additional history revealed previous left carpal tunnel syndrome requiring surgical release followed by carpectomy. His wife also reported gradual coarsening of his facial appearance over several years. On further questioning, the patient recalled progressive increases in both ring and shoe size more than a decade before presentation. These findings appeared disproportionate to the expected manifestations of either systemic sclerosis or transthyretin cardiac amyloidosis, prompting endocrine evaluation.

Review of prior medical records identified an elevated IGF-1 level of 417 ng/mL (reference range: 20-196 ng/mL; Z-score: 3.3 (reference range: -2.0 to +2.0)). Repeat testing confirmed persistent elevation of IGF-1 at 356 ng/mL (reference range: 20-196 ng/mL; Z-score: 2.9), with an elevated growth hormone concentration of 3.7 ng/mL (reference range: 0.05-3.0 ng/mL) and low total and free testosterone levels. Oral glucose suppression testing demonstrated inadequate growth hormone suppression, with a baseline level of 3 ng/mL and a nadir of 2.5 ng/mL; IGF-1 measured at the time was 412 ng/mL (reference range: 34-245 ng/mL). These biochemical findings, together with the clinical phenotype, supported the diagnosis of acromegaly. Pituitary magnetic resonance imaging (MRI) demonstrated a 12×9×7 mm left-sided macroadenoma with inferior extension into the sphenoid sinus. The patient subsequently underwent an uncomplicated transsphenoidal resection in May 2022, with histopathological examination confirming a pituitary adenoma. Follow-up testing demonstrated normalization of IGF-1, consistent with biochemical remission. Despite remission of acromegaly, the cutaneous sclerosis persisted, supporting a distinct coexisting sclerosing process. Serial MRIs in July 2022, August 2023, June 2024, and June 2025 showed no evidence of residual or recurrent tumor. Postoperative IGF-1 levels progressively declined to 185 (Z-score: 1.4) in June 2023 and subsequently to 166 (Z-score: 1.2). At follow-up, cutaneous tightness persisted, and the patient continued to tolerate tafamidis 61 mg daily.

## Discussion

This case illustrates the diagnostic complexity that arises when multiple rare diseases with overlapping clinical manifestations coexist. More importantly, it demonstrates the importance of continually reassessing whether all clinical findings can be explained by a working diagnosis. 

The diagnosis of systemic sclerosis was the most challenging aspect of this case. Skin thickening of both hands extending proximal to the metacarpophalangeal joints met the sufficient criterion for systemic sclerosis classification under the 2013 ACR/EULAR criteria [5]; RNA polymerase III antibody positivity provided additional disease-specific serologic support. However, classification criteria do not substitute for clinical diagnosis, particularly when scleroderma-like disorders are present. The absence of Raynaud phenomenon, antinuclear antibody positivity, digital ulceration, and telangiectasias contributed to an atypical clinical phenotype and warranted careful evaluation for systemic sclerosis mimickers [6].

Morphea was considered based on the initial biopsy interpretation but was less consistent with the rapidly progressive, bilateral, symmetric, and extensive distribution of sclerosis without discrete plaques. Nephrogenic systemic fibrosis was considered less likely because histopathology lacked the expected prominent fibroblastic proliferation. Cutaneous amyloidosis was also evaluated given the subsequent diagnosis of transthyretin cardiac amyloidosis, but Congo red staining did not demonstrate amyloid deposition. Scleroderma mimickers share skin thickening but differ substantially in pathophysiology, distribution, associated systemic findings, and treatment [7].

Acromegaly further complicated the diagnostic evaluation, as chronic growth hormone/IGF-1 excess can cause acral enlargement, soft tissue thickening, coarse facial features, macroglossia, arthropathy, and carpal tunnel syndrome [3,4]. These manifestations, particularly skin thickening and acral enlargement, can overlap with those of scleroderma-spectrum disease, creating additional diagnostic uncertainty. In contrast, the profound and rapidly progressive dermal induration in this patient extended proximally beyond the hands and occurred over a substantially shorter time course than his decade-long acral enlargement. The distribution of sclerosis, sclerotic histopathology, and RNA polymerase III antibody positivity therefore remained difficult to attribute to acromegaly alone. Persistence of cutaneous sclerosis despite biochemical remission of acromegaly further supported a distinct coexisting sclerosing process.

Similarly, transthyretin cardiac amyloidosis accounted for the patient's progressive heart failure symptoms, ventricular hypertrophy, and conduction disease [2]. The combination of characteristic echocardiographic findings, grade 3 technetium-99m pyrophosphate myocardial uptake, and a negative monoclonal protein evaluation is strongly supportive of transthyretin cardiac amyloidosis without requiring tissue biopsy in the appropriate clinical settings [8,9]. Retrospectively, the patient's history of lumbar spinal stenosis and prior carpal tunnel syndrome represented classic extracardiac manifestations that often precede overt cardiac disease by several years [9].

Several clinical findings remained unexplained by systemic sclerosis and transthyretin cardiac amyloidosis alone. Although macroglossia can occur in amyloidosis, its presence alongside coarse facial features and longstanding acral enlargement prompted further endocrine evaluation, leading to the diagnosis of acromegaly secondary to a pituitary macroadenoma. Transsphenoidal resection resulted in biochemical remission, further supporting the diagnosis [10]. 

The coexistence of systemic sclerosis, transthyretin cardiac amyloidosis, and acromegaly created substantial diagnostic ambiguity because of their overlapping manifestations. This case emphasizes the importance of recognizing discordant findings and maintaining diagnostic flexibility when a single disease process does not adequately explain the complete clinical phenotype.

## Conclusions

This case demonstrates the diagnostic complexity of a rapidly progressive scleroderma-like phenotype in a patient with objectively confirmed transthyretin cardiac amyloidosis and acromegaly. This case emphasizes the importance of maintaining a broad differential diagnosis when individual clinical features appear disproportionate to an established diagnosis. Careful evaluation of alternative explanations and continued reassessment of discordant findings can prevent premature diagnostic closure, facilitate recognition of coexisting diseases, and enable timely disease-specific management.
